# Why aren't people with young onset dementia and their supporters using formal services? Results from the INSPIRED study

**DOI:** 10.1371/journal.pone.0180935

**Published:** 2017-07-19

**Authors:** Monica Cations, Adrienne Withall, Ruth Horsfall, Nicole Denham, Fiona White, Julian Trollor, Clement Loy, Henry Brodaty, Perminder Sachdev, Peter Gonski, Apo Demirkol, Robert G. Cumming, Brian Draper

**Affiliations:** 1 School of Public Health and Community Medicine, UNSW Australia, Syndey, NSW, Australia; 2 Dementia Collaborative Research Centre–Assessment and Better Care, School of Psychiatry, UNSW Australia, Sydney, NSW, Australia; 3 Centre for Healthy Brain Ageing, UNSW Australia, Sydney, NSW, Australia; 4 Department of Developmental Disability Neuropsychiatry, School of Psychiatry, UNSW Australia, Sydney, NSW, Australia; 5 Sydney School of Public Health, The University of Sydney, Sydney, NSW, Australia; 6 Academic Department for Old Age Psychiatry, Prince of Wales Hospital, Randwick, NSW, Australia; 7 Neuropsychiatric Institute, Prince of Wales Hospital, Randwick, NSW, Australia; 8 Division of Aged and Extended Care (Southcare), Sutherland Hospital, South Eastern Sydney Local Health District, Sydney, NSW, Australia; 9 The Langton Centre, South Eastern Sydney Local Health District, Drug and Alcohol Services, Surry Hills, NSW, Australia; Taipei Veterans General Hospital, TAIWAN

## Abstract

**Background/Aims:**

Despite reporting high levels of burden, supporters of people with young onset dementia (YOD) underuse formal community services. Previous quantitative studies in YOD are of limited utility in guiding service design because they did not consider important contextual barriers to service use. The aim of this study was to identify all relevant barriers and describe the service features considered most important to improving uptake by people with YOD and their supporters.

**Methods:**

Eighty-six people with consensus-confirmed YOD (mean onset age 55.3 years) and/or their primary supporter participated in quantitative interviews, and 50 also participated in one of seven qualitative focus groups. Interview participants reported levels of community service use and reasons for non-use, functional impairment, behavioural and psychological symptoms, supporter burden, social network, and informal care provision. Focus group participants expanded on reasons for non-use and aspects of an ideal service.

**Results:**

Although at least one community service was recommended to most participants (96.8%), 66.7% chose not to use one or more of these. Few of the clinical or demographic factors included here were related to service use. Qualitative analyses identified that lack of perceived need, availability, and YOD-specific barriers (including ineligibility, unaffordability, lack of security, lack of childcare) were commonly reported. Five aspects of an ideal service were noted: unique, flexibile, affordable, tailored, and promoting meaningful engagement.

**Conclusion:**

People with YOD and their families report that formal community services do not meet their personal and psychological needs. Researchers can provide ongoing assessment of program feasibility, suitability, and generalisability.

## Introduction

Young onset dementia (YOD; onset of symptoms under the age of 65) is associated with significant distress and burden [1,2]. Contributing factors include emotional and financial strain when work is reduced or ceased prematurely by both the person with YOD and their primary supporter, and the difficulties of balancing competing care needs of young children and older parents [3–6]. Use of formal community care services can delay institutionalisation [7], provide respite [8], facilitate access to peer support [9], and spread the caregiving load [10]. Despite these benefits, previous studies have identified a low level of formal care use in YOD groups [11–14].

Possible barriers and enablers to service use in YOD are not well understood, with limited quantitative evidence available to guide service design. Most studies have that have established poor uptake of services did not explore possible reasons for this [13–15]. Anderson [16] posits that the strongest predictor of formal service use in any context is perceived need, and this appears to apply well to older people with dementia and their supporters [17]. Whether this is also true for YOD is not clear, particularly considering that in qualitative studies people with YOD and their supporters have expressed dissatisfaction with services that are tailored to the needs of older people [18,19]. Establishing the experiences of people with YOD and their supporters, and their perceived barriers to service use, can inform design and in turn improve uptake of the services intended for them [20].

Bakker et al. [11] assessed the impact of clinical factors and found that service use was only tenuously associated with the person’s severity of functional and cognitive impairment, such that many supporters would access help only when care needs inevitably became greater than they could provide. However, this study did not consider the importance of contextual or service-specific barriers to use. In addition, previous studies have not examined individual service types separately to establish specific reasons for use or non-use, instead adopting a ‘total score’ approach in their analyses [8].

This mixed-methods study explores both demographic/clinical and contextual/service-related barriers and enablers to use of different types of formal community services in YOD. The aim is to determine the most common barriers to service use and describe the service features considered most important to improving uptake by people with YOD and their supporters. This information is used to establish recommendations for YOD service design.

## Methods

The ‘Improving Service Provision for Younger Onset Memory and Related Disorders’ (INSPIRED) study is a cross-sectional study of YOD epidemiology in New South Wales (NSW), Australia. A detailed description of recruitment appears elsewhere [21]. Briefly, people with YOD and/or their supporters from across metropolitan Sydney and the South Coast of NSW were invited to participate via health professionals, service providers and general advertising. Participants were included if the onset of dementia symptoms occurred before age 65 and their diagnosis could be confirmed via clinical consensus. With informed written consent, participants and/or their primary supporter completed a structured interview on a rolling basis from September 2011 to May 2014.

A subset of INSPIRED participants was included in one of seven focus groups conducted between November 2012 and July 2015. Recruitment for focus groups occurred concurrently with recruitment for interviews, and all participants who contacted the study team were invited for both. People with YOD and primary supporters were both invited, though one was included even if the other declined. There were no demographic exclusion criteria and all consenting participants were included. In some cases, participants opted to take part in a focus group in lieu of a study interview. Groups were intentionally varied by age, sex and dementia type to provide a breadth of perspectives. Each group provided written consent for audiotaping and these were transcribed verbatim. Focus groups were between 60 and 100 minutes in duration, were facilitated by study staff, with four to eight participants included in each. Participants indicated a preference for the term ‘supporter’ and as such this terminology is used in place of ‘carer’ or ‘caregiver’ here.

### Measures

A structured interview guide was used to collect quantitative data and included demographic information, formal service use (yes vs no, grouped by service type), five validated scales measuring functional impairment, behavioural and psychological symptoms of dementia (BPSD), supporter burden and isolation, and caregiving time (see ‘Demographic and clinical factors’ section below) and an open ended question for each service type regarding reason for non-use, if relevant. This information was used to develop open-ended questions regarding barriers and enablers to service use that were explored in more depth in qualitative focus groups.

#### Demographics

In addition to demographic details collected at interview, details regarding dementia diagnosis were obtained from the clinical consensus process [21]. Time spent caregiving (as primary and direct supporter) was calculated as months between the participant’s age at symptom onset and age at interview or at placement in residential care.

#### Formal community service use

Participants were presented with a comprehensive list of formal community care services compiled by study investigators and asked to indicate whether they had been recommended the service or used the service at any time since their diagnosis (including before residential care placement). Services fell into four categories: respite (e.g. day care programs, residential respite, community activity programs), psychosocial support or psychoeducation (e.g. education and support groups, behaviour change advisory services, dementia helpline, mental health services), allied health (e.g. speech pathology, neuropsychology, physiotherapy), and in-home care (e.g. Meals on Wheels, cleaning or gardening services, community care financial packages, domiciliary care, social visitors). Residential care placement was not counted as community service use. All of these services are available in Australia but accessibility varies widely. Most are provided via the aged care sector and subsidised by Commonwealth aged care funding (particularly respite and in-home care services). Psychoeducation and advisory services are delivered by not-for-profit organisations such as Alzheimer’s Australia and by aged health services. Allied health services are located in both public health and private sectors, with the latter having some subsidies in Medicare and private health insurance.

#### Demographic and clinical factors

Severity of functional disability was assessed during interview using the 12-item interview version of the World Health Organisation Disability Assessment Schedule II (WHODAS; [22,23], in which respondents are asked to rate difficulty in the past 30 days with a range of cognitive and functional tasks and a total percentage of disability is calculated. This scale has very high internal consistency (α = 0.90–0.97) [24] and performs well when discriminating varying levels of disability [25].The 45-item Cambridge Behavioural Inventory-Revised (CBI-R) [26] was used to assess behavioural symptoms. Supporters rated the frequency of behaviours across 10 domains for a total possible score of 180. The CBI-R has the capacity to discriminate between dementia types and individual items correlate with the original scale (α = 0.58–0.93) [26]. Participants who died prior to interview were not included in analyses using these outcomes.

Subjective supporter burden was measured using the shortened 12-item version of the Zarit Burden Interview [27], summed to a maximum score of 48. This shortened version correlates well with the original 21 and 22-item versions [27] and has high internal consistency (α = 0.87) and discriminant validity (AUC = 0.99) [28]. The social network of the primary supporter was assessed using the abbreviated 6-item version of the Lubben Social Network Scale [29]. This scale asks supporters to report on the number of family members and friends with whom they speak regularly, can discuss private matters and can call on for help (range 0–30). It is noted to have a homogenous factor structure and adequate internal consistency (α = 0.83) [29]. Provision of informal care was assessed using the Resources Utilisation in Dementia-Lite (RUD-Lite) questionnaire [30], in which participants were asked to report the total number of hours spent providing care in the past month. The RUD-Lite covers 95 per cent of items in the original RUD, but is less onerous. Estimated caregiving time on the original RUD compares well to direct observation (*r* = 0.74–0.93) [31].

#### Barriers and enablers to service use

In cases where the participants specified that a service had been recommended to them but never used they were asked to report the primary reasons for non-use. This question was open-ended but prompts were used where required. Responses were then categorised into groups using an inductive approach. Monica Cations read all responses and coded them into related concepts, which were then reviewed for important sub-themes and new codes created. Codes were reviewed by co-authors Brian Draper and Adrienne Withall to ensure all relevant responses were captured and that similar themes were grouped.

Barriers and enablers to service use were further explored in focus groups. Participants were asked to reflect on their experiences from symptom onset to the present time. Question prompts relevant to this study were: a) Across all stages of your illness, what services were needed the most/what would have been helpful? What were the barriers to accessing these? What were the enablers? and; b) What are your concerns for the future? Themes identified were used to guide development of recommendations for service design.

### Statistical analysis

The outcome for the quantitative analyses for this study was non-use of a recommended service (yes/no). A small amount of missing data (<4%) was noted, but Little’s test [32] found no systematic pattern among this and multiple imputation was used to complete the dataset. Logistic regression modelling was conducted in statistical program SPSS (v.22) [33] to assess the effect of selected demographic and clinical factors on service use. Analyses were repeated excluding participants deceased or living in residential aged care to establish ‘current’ barriers. Contextual/service-related barriers to use are displayed descriptively.

Transcripts of audio recordings from the focus groups were thematically analysed using an inductive approach [34] with interaction between data collection and analysis. That is, themes identified early in data collection informed subsequent data collection. Recurrent themes were compared across participants and groups, and attention was paid to ‘negative cases’ where a different view to most respondents was discovered that may indicate an alternate view [34]. The group (rather than the individual responses) is most critical in focus group thematic analysis [35], and as such the number of groups that discussed a theme or subtheme was given greater consideration than the individual responses. Qualitative data were managed using NVIVO v.10 [36].

### Ethics

This study was approved by the Human Research Ethics Committees (Health and Medical) of the University of Wollongong, University of NSW and the South Eastern Sydney and Illawarra Area Health Service.

## Results

Of 93 participants recruited for INSPIRED, three were diagnosed with MCI during clinical consensus and were excluded from the current analyses. Four cases of confirmed YOD were also excluded as insufficient detail was available regarding their service use. There were no differences in age, sex, urban/regional distribution or dementia type between included and excluded participants.

The final sample for the quantitative analysis included 86 people with a clinically confirmed diagnosis of YOD, of whom 64 were interviewed together with their supporter. One participant lived alone and was interviewed alone. The remaining 21 participants with YOD were either too cognitively impaired (*n* = 19) or died after recruitment but prior to interview (*n* = 2) and in these cases supporters provided all information on their behalf. Demographic and clinical information is presented in Table 1. The subset of participants in the qualitative analysis included 40 supporters and 10 people with YOD across the seven focus groups, providing adequate saturation for thematic analyses. Thirty-nine of these (including all people with YOD) had completed an INSPIRED interview while 11 supporters participated in a focus group in leiu of an interview. Dementia aetiology in focus group participants included AD (*n =* 6), FTD (*n =* 2), LBD (*n =* 1) and mixed dementia (*n* = 1).

**Table 1:** Sample demographics.

		*n* (%) or ẋ (SD)		
		All interview participants (*n* = 86)	Community dwelling interview partiicpants (*n* = 65)	Interview participants in RACF (*n* = 19)^b^
Participants with YOD (*n* = 86)				
Age at interview		62.9 (6.2) R: 45–79	60.8 (8.8) R: 45–72	66.5 (7.2) R: 57–79
Age at onset		55.3 (6.0) R: 35–64	55.1 (6.1) R: 35–64	56.6 (5.2) R: 47–64
Male		61 (70.9)	45 (70.8)	13 (68.4)
Years of education		13.2 (4.2)	13.3 (3.6)	11.8 (4.2)
English as a second language		14 (16.3)	10 (15.4)	4 (21.1)
Dementia type	Alzheimer’s disease (AD)	47 (54.7)	37 (56.9)	9 (47.4)
	Frontotemporal dementia (FTD)	14 (16.3)	9 (13.9)	5 (26.3)
	Unspecified dementia	6 (7.0)	3 (4.6)	3 (15.8)
	Mixed dementias	5 (5.8)	3 (4.6)	1 (5.3)
	Vascular dementia (VaD)	5 (5.8)	5 (7.7)	0 (0)
	Progressive supranuclear palsy	4 (4.7)	4 (6.2)	0 (0)
	Dementia in Huntington’s disease	3 (3.5)	3 (4.6)	0 (0)
	Lewy Body Disease (LBD)	2 (2.3)	1 (1.5)	1 (5.3)
Care arrangements (at interview)	No supporter	1 (1.2)	1 (1.5)	0 (0)
	Living with supporter	64 (74.4)	64 (98.5)	0 (0)
	Residential Aged Care Facility	19 (22.1)	N/A	19 (100)
	Deceased	2 (2.3)	N/A	0 (0)
WHO Disability Assessment Schedule		0.57 (0.3)	0.50 (0.2)	0.77 (0.2)
Cambridge Behaviour Inventory—Revised		52.1 (31.4)	46.3 (22.1)	56.7 (30.4)
Supporters (*n* = 85)				
		Whole sample (*n* = 85)	Living with participant (*n* = 64)	Not living with participant (*n* = 21)
Age at interview		60.2 (9.0) R: 31–77	59.9 (8.0) R: 33–74	61.2 (11.6) R: 31–77
Male		21 (24.4)	17 (26.6)	4 (19.1)
Supporter years of education		13.3 (3.9)	13.2 (4.1)	13.1 (4.6)
Relationship to participant with YOD	Spouse / partner	71 (82.6)	57 (89.1)	14 (66.7)
	Sibling	4 (4.7)	1 (1.6)	3 (14.3)
	Son / daughter	4 (4.7)	1 (1.6)	3 (14.3)
	Parent	3 (3.5)	2 (3.2)	1 (4.8)
	Friend / neighbour	3 (3.5)	3 (4.7)	0 (0)
Time spent caring (months)		81.4 (59.2)	78.3 (56.7)	123.6 (46.7)
Ever lived with participant with YOD		82 (95.3)	N/A	20 (95.2)
Employment	Not in the work force	40 (47.1)	29 (44.6)	12 (57.2)
	Part time	24 (28.3)	19 (29.2)	3 (14.3)
	Full time	21 (24.7)	16 (24.6)	6 (28.6)
Working hours (per week)		13.32 (3.9)	15.5 (8.0)	17.2 (21.9)
Zarit Burden Interview		12.3 (8.0)^a^	12.8 (7.3)	10.6 (9.5)^a^
Lubben Social Network Scale		15.8 (6.5)^a^	15.9 (5.7)	15.6 (8.4)^a^
Hours of informal care in previous month (*n* = 64)		351.6 (338.9)	348.4 (340.6)	0 (0)

^a^*n* = 83, excluding supporters of two deceased participants with dementia

^b^Excludes 2 people with YOD who died prior to interview

R = Range; SD = standard deviation; YOD = Young onset dementia

### Formal service use

Rates of formal service use are presented in Table 2. Knowledge of services was overall very high, with nearly all participants (96.5%) recommended at least one formal service. However, two in three participants (66.3%) chose not to use at least one formal service that was recommended to them.

**Table 2:** Formal service use.

	Recommended *n* (%)			Used *n* (%)		
	Whole sample (*n* = 86)	Community-dwelling (*n* = 65)	RACF and deceased (*n* = 21)	Whole sample (*n* = 86)	Community-dwelling (*n* = 65)	RACF and deceased (*n* = 21)
Any formal service	83 (96.5)	62 (95.4)	20 (95.2)	80 (93.1)	61 (93.8)	19 (90.5)
Respite service	72 (83.7)	52 (80.0)	20 (95.2)	52 (60.5)	34 (52.3)	18 (85.7)
Psychoeducation service	74 (84.9)	60 (92.3)	14 (66.7)	51 (59.3)	42 (64.6)	9 (42.9)
Allied health service	48 (55.9)	34 (52.3)	10 (47.6)	26 (30.1)	18 (27.7)	8 (38.1)
In-home care service	58 (67.4)	45 (69.2)	13 (61.9)	41 (47.7)	32 (49.2)	9 (42.9)

RACF = Residential aged care facility

Few of the demographic or clinical factors studied here were associated with formal service use (Table 3). More severe BPSD and a non-Alzheimer dementia were both associated with increased service uptake, though this effect did not retain significance when non-community dwelling participants were removed from the analysis. Socially isolated supporters were less likely to use formal services.

**Table 3:** Univariate logistic regression results–selected demographic and clinical factors associated with formal care use.

	Whole sample (*n* = 86)		Community-dwelling (*n* = 65)	
	OR (95% CI)	*p*	OR (95% CI)	*p*
Participant age	0.98 (0.92–1.36)	0.39	0.91 (0.81–1.02)	0.09
Participant sex	1.04 (0.39–2.81)	0.95	0.99 (0.30–3.33)	0.99
Participant dementia type^a^	**0.36 (0.15–0.90)**	**0.03**	0.42 (0.14–1.30)	0.14
Supporter age	0.99 (0.94–1.04)	0.61	0.99 (0.92–1.07)	0.75
Supporter sex	1.79 (0.71–4.55)	0.23	2.02 (0.60–6.81)	0.26
Supporter working hours^b^		N/A	1.02 (0.99–1.06)	0.39
Supporter time spent caring	1.01 (1.00–1.02)	0.36	1.02 (0.99–1.03)	0.07
Zarit total^b^		N/A	1.01 (0.94–1.09)	0.87
Lubben Total^b^		N/A	**1.07 (1.10–1.18)**	**0.02**
WHODAS Total	0.18 (0.03–1.13)	0.07	0.46 (0.04–5.59)	0.55
CBI-R Total	**0.99 (0.97–0.99)**	**0.04**	1.00 (0.98–1.03)	0.09
Informal care hours^b^		N/A	1.00 (0.99–1.00)	0.13

^a^AD vs Other

^b^Based on *n* = 64 supporters currently providing care; whole sample analyses not relevant.

CI = confidence interval; CBI-R = Cambridge Behavioural Inventory-Revised; N/A = not applicable; OR = odds ratio; WHODAS = World Health Organization Disability Assessment Schedule

### Qualitative findings

All interview participants reported one or more contextual or service-related reasons for not using a service (Table 4). These reasons could be categorised into four groups: (1) no perceived need for service; (2) participant or supporter refusal; (3) service not available or affordable, and; (4) YOD-specific reasons. YOD-specific reasons included that the service was only eligible to those over 65 years old, did not provide adequate security for young and physically agile participants, required a superannuation or pension co-payment, or did not provide adequate child care. Service-related barriers often intersected with personal factors, typified by the negative psychological impact when a service was designed for and dominated by older users.

**Table 4:** Reasons for service non-use.

	Whole sample (n = 86)					Community-dwelling (n = 65)				
	Recommended but not used *n* (%)	Reason for non-use *n* (%)				Recommended but not used *n* (%)	Reason for non-use *n* (%)			
		Service not needed	PwD / Supporter Refusal	Service not available	YOD-related reason		Service not needed	PwD / Supporter Refusal	Service not available	YOD-related reason
Any formal service	57 (66.3)	23 (40.4)	21 (36.9)	22 (38.6)	28 (49.2)	48 (73.8)	20 (41.7)	18 (37.5)	19 (39.6)	21 (43.8)
Respite service	20 (23.3)	7 (35.0)	6 (30.0)	7 (35.0)	7 (35.0)	18 (27.7)	7 (38.9)	6 (33.4)	6 (33.4)	6 (33.4)
Psychoeducation service	23 (26.8)	4 (17.4)	5 (21.8)	6 (18.8)	11 (47.8)	18 (27.7)	4 (22.3)	3 (16.7)	4 (22.3)	7 (38.9)
Allied health service	22 (25.6)	7 (33.3)	3 (14.3)	6 (28.6)	8 (38.1)	16 (24.6)	5 (31.3)	2 (12.5)	5 (31.3)	4 (25.0)
In-home care service	17 (19.8)	14 (82.4)	3 (17.7)	10 (58.9)	8 (47.1)	13 (20.0)	12 (92.3)	0 (0.0)	8 (61.6)	5 (38.5)

PwD = person with dementia; YOD = young onset dementia

Barriers and enablers to service use were explored in more depth in focus groups. The broad themes common to all focus groups were:

- appropriateness of services,
- timely access to services, knowledge and information,
- case management, key workers and service network access,
- meaningful social connections, activities and support groups,
- community awareness and stigma, and
- transition to care

#### Appropriateness of services

A major theme concerned the appropriateness of services as participants noted dissatisfaction with YOD programs being offered via aged care, and having difficulty relating to older peers.

“I went to the original Alzheimer’s group down in my area and everybody’s nearly 30 years older than me. So they’re all talking about pensions and those sorts of things… There was nobody my age.”

Being included with much older counterparts created a mismatch in both physical abilities and leisure interests, which a number of participants noted had a significantly negative psychological impact. Supporters of people with YOD who lived or attended respite in residential care also noted security concerns, as physically agile participants could easily navigate security systems and abscond.

#### Access to services

Access to services was recognised as a problem beyond the lack of relevant services available. It was apparent that this often started very early, during diagnosis, and was further exacerbated by delays in access to funding assessments or ineligibility due to age.

“Getting that diagnosis and then being put back in the waiting room and not even made another appointment, not told what to do. We’re in shock, and then we had to drive home… I don’t think we spoke all the way home.”

A variety of access barriers were noted, including to transport, services outside of work time, and concurrent care for dependents. Financial barriers were common, particularly in the context of having to cease work many years prior to the eligibility age for superannuation or pensions. This limited access to services at all stages, from diagnostic specialists through to residential care placement. While some financial support was available, participants noted that these were very modest and often involved a lengthy and confusing bureaucractic process.

“They weren’t going to pay his super—no way were they going to give us that. But we actually ended up realising that he had income protection… But, every six months we have to go back and jump through all the hoops, and go to the doctors and get all the reports…”

#### Knowledge and information

Strong themes emerged regarding the lack of access to the right information, at the right time and in the right way. While some participants reported receiving no information, others reported information overload with no help to wade through it. When available, YOD specific psychoeducation proved an excellent source of information.

“We did the early onset course, that was incredibly helpful. I don't think it could have been more helpful. It really set us off on the journey.., I just don't know how you would have coped without it.”

#### Case management

There was very strong support in all groups for a case management approach to YOD that would provide a central information and referral point referred to as a ‘one-stop shop’. Many advocated for referral to be the responsibility of the diagnosing specialist. A key worker model was introduced and funded by the Federal Government toward the end of this study. This role was distinct from a case manager insofar that there was no automatic referral process and the key worker could not recommend or refer to particular services. They instead provided information regarding a range of options and could link the client with a service upon request. Nonetheless, participants who had accessed the service reported great satisfaction with it:

[The key worker] sort of had all the–the support that we needed. The means to, to open those doors, I suppose. To give us the information that we needed. Otherwise, there was, there was really nothing—nothing at that stage.

#### Social connections

Programs aimed at alleviating social isolation were praised. Participants found that friends and family had difficulty understanding and coping with the disease and at times avoided contact. Support groups played an important role in alleviating social isolation for both people with YOD and supporters. They were also a valuable source of information, with supporters noting a snowball effect of each service improving access to the next

“Meeting other people is what helps me, it’s so important. …Nobody else, none of my friends, are dealing with this.”

The content of social groups for people with YOD was discussed. Many participants noted the desire for meaningful engagement that extends beyond simply being occupied. An ‘ideal’ program was individualised and delivered in small groups, supporting the person with YOD to keep working or otherwise contributing to the community. Engaging in important pre-diagnosis activities was an important outcome.

#### Transition to care

Finally, supporters of people with YOD now living in residential care noted a need for support to manage this transition, citing concerns about the suitability and security of facilities, staff reluctance to accept younger residents, and the emotional impact of the move much earlier in life than was expected.

## Discussion

The key finding from this mixed-methods study is that barriers to formal service use in YOD are unique and reflect the complexities associated with dementia onset in midlife. A mismatch is evident in that services are offered under the banner of aged (or geriatric) care to participants who are not yet eligible or suitable for this system. This causes reluctance to use services even when they are recommended, and dissatisfaction with services when they are used.

### Main findings

Both knowledge of services (96.5%) and rates of service use (93.1%) were overall very high in this study, and higher than in previous studies [11,13–15,37]. This may be related to improvements in diagnosis and service pathways for YOD over time, but the sampling method is also a likely contributor. As all participants were recruited via their health care professional or advertising with community dementia services, awareness and service use may be overestimated here.

Despite this, a significant majority of participants (66.3%) did not engage with at least one formal service that was recommended to them. Quantitative analysis of demographic or clinical factors related to service use were perhaps underpowered (especially given the high rates of service use), but did suggest a tenuous relationship. Both greater severity of BPSD and dementia type were associated with service uptake, but only when participants living in full time residential care (with very high care needs) were included in the analysis. This finding is congruent with those from Bakker et al. [11] suggesting that service use may be delayed until impairment is very severe (though could also reflect a drop in statistical power). Supporter social isolation was also related to service non-use in univariate analysis, but exploration of this finding in focus groups suggested that reduced isolation was a positive consequence of service use rather than the other way around. Many supporters reported a snowball effect of service use in which engagement with each service improved social support and access to the next service.

Qualitative exploration of barriers to service use indicated that perceived need, limited accessibility of the service, and barriers specific to YOD were all relevant and their impact extended across all service types. That perceived need contributes to service use in YOD is not surprising. It has been demonstrated as the most powerful predictor of service use in a variety of contexts, including older people with dementia [16,17]. Authors of one study that compared service use in YOD to an older sample noted that YOD supporters felt better prepared to provide care and were better informed about support services, crediting both better supporter health and internet literacy in this cohort [38]. For participants with dementia, lack of perceived need may also reflect low insight about the severity of their symptoms.

The contribution of YOD-specific barriers identified in this study is an important insight for service designers. A variety of factors unique to this younger population were identified in quantitative interviews, and these were discussed at length in all qualitative focus groups. These ranged from practical concerns (such as financial barriers or lack of available childcare) to concerns about the psychological impact of being placed in programs with much older peers (or the combination of these). Focus group participants described a sense of indignity and ‘otherness’ that placement with older participants adds to an already difficult journey, and this theme has been referenced in earlier qualitative studies [5,18]. Financial difficulties were commonplace and were compounded with frustration at navigating complex bureaucractic processes.

### Recommendations for service design

The results of this study and particularly focus group themes were used to develop recommendations for service design. Five core elements of a ‘good’ service for people with YOD were clear:

1. Unique
  Services offered to people with YOD and their supporters should be designed with their unique circumstances in mind, and should be separate from those offered to older people where possible.
2. Tailored and timely
  Service needs vary over time and are unique to the person and their family. The provision of a specialised case manager that is accessible from the time of diagnosis was widely supported by our sample. The key worker role introduced in Australia was valued very highly by study participants who had accessed it. Of course, the availability of services and programs to which these case managers can refer are required in order for this model to be effective.
3. Financially accessible
  Frequent reference was made in this study to the cost-prohibitive nature of available services. Service designers should consider the financial strains and constraints associated with YOD and make use of volunteers and platforms that are cost-effective. Particular support should be offered to help people with YOD and their families to access financial aid and/or planning where available.
4. Flexible
  People with YOD and their families require innovative methods of service delivery based on their complex occupational and other care arrangements. Services that are offered outside of business hours and/or on the weekend may be necessary. The potential value of online programs is recognised for this cohort given their pre-morbid computer literacy. Piloting is underway in the Netherlands for an online psychoeducation program for YOD supporters [39], and support groups on social media are increasingly popular [40].
5. Meaningful
  Finally, focus group participants promoted the need for meaningfully or purposefully engaging activity that extends beyond keeping occupied. Some programs that support ongoing employment or volunteer work have been trialled, but outcomes have not been formally measured [41]. Nonetheless, projects like these are gaining attention among YOD advocates who call for active rehabilitation programs similar to those offered in other neurological conditions [42]. Despite the degenerative nature of dementia, multidisciplinary rehabilitation may prolong functional independence [43] and is worth piloting in this population.

### Limitations

Results of this study should be interpreted in the context of methodological limitations. The sample for this study is small and may have been underpowered to identify subtle effects. Statistical corrections to account for the number of tests were not possible. As previously mentioned, participants were recruited via health professionals or advertising with service providers, and population service use may be overestimated. The diversity of the sample also bears comment. Participants were all metropolitan or inner regional-dwelling, with none living in outer regional or remote areas. Only one person with YOD lived alone and participants from diverse social and cultural backgrounds were under-represented. This is problematic considering that the suitability of services in older populations is highly dependant on these demographic factors [44], and may be another reason why service use was so high in the current sample. The use of mixed focus groups that included a majority of supporters may also have skewed the qualitative findings, under-representing the views of people with dementia. The recommendations may reflect the needs of supporters more than those they care for. Finally, this is a cross-sectional study with no control group of older people with dementia, limiting the ability to compare barriers to service use across groups or over time.

### Future directions

Results of the current study confirm what has been reported qualitatively [19,45–48] by people with YOD and their supporters for some time. The need for flexible, accessible and tailored YOD services is clear. How to ensure these services are cost-effective in the context of a low prevalence disorder spread across a large geographical landscape remains a challenge. Researchers can assist with this process by thoroughly piloting program feasibility, accessibility and cost-effectiveness, and conducting implementation studies that are evaluated across a variety of diverse populations. Comparison of the YOD service experiences and needs of people from different geographical, cultural and social contexts is also needed to understand barriers specific to these groups.

## Conclusions

This mixed-methods study has shown that people with YOD and their families choose not to use services that are recommended to them for a variety of reasons that are unique to the experience of dementia in midlife. Participants reported that services did not meet practical or psychological needs, and were dissatisfied with assimilating into programs designed for those many years their senior. A number of aspects of ‘good’ services were identified, most importantly that the services be unique, flexibile, affordable, tailored, and promote meaningful engagement. Service designers will benefit from ongoing research assessing program feasibility, suitability, and generalisability to diverse populations.

## Supporting information

S1 File Anonymised minimum data set for quantitative data. (XLSX)
